# Peritoneal Dialysis-Related Peritonitis Due to *Staphylococcus aureus*: A Single-Center Experience over 15 Years

**DOI:** 10.1371/journal.pone.0031780

**Published:** 2012-02-21

**Authors:** Pasqual Barretti, Taíse M. C. Moraes, Carlos H. Camargo, Jacqueline C. T. Caramori, Alessandro L. Mondelli, Augusto C. Montelli, Maria de Lourdes R. S. da Cunha

**Affiliations:** 1 Departamento de Clínica Médica, Faculdade de Medicina, UNESP - Universidade Estadual Paulista, Botucatu, São Paulo, Brazil; 2 Departamento de Microbiologia e Imunologia, Instituto de Biociências, UNESP - Universidade Estadual Paulista, Botucatu, São Paulo, Brazil; National Institutes of Health, United States of America

## Abstract

Peritonitis caused by *Staphylococcus aureus* is a serious complication of peritoneal dialysis (PD), which is associated with poor outcome and high PD failure rates. We reviewed the records of 62 *S. aureus* peritonitis episodes that occurred between 1996 and 2010 in the dialysis unit of a single university hospital and evaluated the host and bacterial factors influencing peritonitis outcome. Peritonitis incidence was calculated for three subsequent 5-year periods and compared using a Poisson regression model. The production of biofilm, enzymes, and toxins was evaluated. Oxacillin resistance was evaluated based on minimum inhibitory concentration and presence of the *mec*A gene. Logistic regression was used for the analysis of demographic, clinical, and microbiological factors influencing peritonitis outcome. Resolution and death rates were compared with 117 contemporary coagulase-negative staphylococcus (CoNS) episodes. The incidence of *S. aureus* peritonitis declined significantly over time from 0.13 in 1996–2000 to 0.04 episodes/patient/year in 2006–2010 (*p* = 0.03). The oxacillin resistance rate was 11.3%. Toxin and enzyme production was expressive, except for enterotoxin D. Biofilm production was positive in 88.7% of strains. The presence of the *mec*A gene was associated with a higher frequency of fever and abdominal pain. The logistic regression model showed that diabetes mellitus (*p* = 0.009) and β-hemolysin production (*p* = 0.006) were independent predictors of non-resolution of infection. The probability of resolution was higher among patients aged 41 to 60 years than among those >60 years (*p* = 0.02). A trend to higher death rate was observed for *S. aureus* episodes (9.7%) compared to CoNS episodes (2.5%), (*p* = 0.08), whereas resolution rates were similar. Despite the decline in incidence, *S. aureus* peritonitis remains a serious complication of PD that is associated with a high death rate. The outcome of this infection is negatively influenced by host factors such as age and diabetes mellitus. In addition, β-hemolysin production is predictive of non-resolution of infection, suggesting a pathogenic role of this factor in PD-related *S. aureus* peritonitis.

## Introduction

Peritonitis is a serious complication of peritoneal dialysis (PD) and is responsible for a high rate of technique failure and death in PD patients [1]. Gram-positive cocci are the main etiological agents of peritonitis in the world, with coagulase-negative staphylococci (CoNS) being the most common microbial agents, whereas *Staphylococcus aureus* is associated with more severe episodes, a higher risk of hospitalization, catheter removal, and death [1], [2]. Although *S. aureus* is responsible for a small proportion of peritonitis episodes in most countries, it continues to be the leading cause of this infection in some Latin American countries, particularly in Brazil [3].

A poor prognosis of PD-related *S. aureus* peritonitis has been frequently reported [2], [4], [5], but there are only two reports [6], [7] that specifically describe the clinical outcome and predictors of treatment response in this infection. In the largest series, Govindarajulu et al. [6] showed that methicillin-resistant *S. aureus* (MRSA) peritonitis was independently predictive of an increased risk of permanent hemodialysis transfer and tended to be associated with a high risk of hospitalization. Szeto et al. [7] reported a lower primary response rate and complete cure rate for episodes caused by MRSA compared to episodes due to other *S. aureus*. In both cases the clinical outcome of *S. aureus* peritonitis was not encouraging. The rates of relapse, catheter removal and hospitalization were 20%, 23% and 67%, respectively, in the study of Govindarajulu et al. [6]. In the series of Szeto et al. [7], only 51% of patients with methicillin-susceptible *S. aureus* peritonitis and 46% with MRSA peritonitis presented complete cure without relapse, recurrent or repeat episodes, or need for catheter removal.

In addition to antibiotic resistance, the severity of *S. aureus* infections is associated with virulence factors produced by this bacterium, such as enzymes (coagulase, lipase, and nucleases) and multiple toxins with diverse activities. One family of protein toxins are the staphylococcal enterotoxins and the related toxic shock syndrome toxin-1 (TSST-1) that act as superantigens [8]. The biofilm produced by most *S. aureus* strains facilitates bacterial adhesion to catheters and colonization and simultaneously worsens the response to infection, protecting bacterial cells from the host's natural defense mechanisms and from the action of antibiotics [8], [9]. Although these products may influence clinical outcome, their role in PD-related *S. aureus* peritonitis is still not fully defined. Data published by Haslinger-Löffler et al. [10] suggest that α-hemolysin plays a specific role in the pathogenesis of peritonitis. Using cultured human peritoneal mesothelial cells, these authors showed that α-hemolysin produced by *S. aureus* was able to induce caspase-independent cell death. In a recent study, our group demonstrated that biofilm and α-hemolysin production were the only independent predictors of non-resolution of staphylococcal peritonitis [11]. However, the small number of *S. aureus* episodes analyzed was an important limitation of that study.

For the last 15 years we have monitored clinical and microbiological characteristics of *S. aureus* peritonitis in PD patients, including virulence factors produced by this pathogen and presence of the *mec*A gene that confers resistance to methicillin/oxacillin. The objective of the present study was to describe the experience of a single Brazilian center with PD-related *S. aureus* peritonitis, focusing on host and bacterial factors that influence peritonitis outcome.

## Results

**Figure 1:**
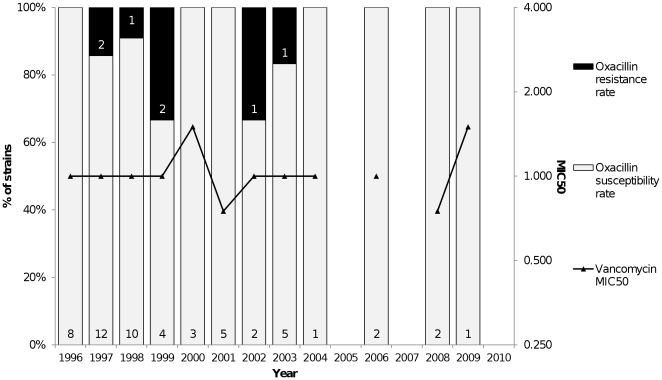
Annual Rate (episodes/patient/year) of *Staphylococcus aureus* Peritonitis from January 1996 to December 2010.

A total of 682 peritonitis episodes were diagnosed in our unit between 1996 and 2010. The overall peritonitis rate was 0.96 episodes per patient per year. Seventy-three (10.7%) episodes were caused by S. *aureus*. After application of the exclusion criteria, 62 episodes that occurred in 56 patients were analyzed. The demographic and baseline clinical data of the patients are summarized in Table 1. The clinical findings in peritonitis episodes were expressed in Table 2. The incidence of *S. aureus* peritonitis declined significantly over time and was 0.13 episodes per patient per year in 1996–2000, 0.10 in 2001–2005, and 0.04 in 2006–2010 (*p* = 0.03). The annual *S. aureus* peritonitis rate is presented in figure 1; a strong decline of the incidence was observed after 2003. Vancomycin was used in 35 (56.5%) episodes. Overall, 32 (51.6%) episodes were resolved; among cases that were not resolved one (0.16%) relapsed, 18 (29%) required removal of the catheter due to refractory peritonitis, five (8%) were resolved with a second antibiotic regimen, and six (9.7%) progressed to death. Of 117 contemporary CoNS peritonitis episodes, 63 (53.8%) were resolved and three (2.5%) progressed to death. The death rate tended to be lower among episodes caused by CoNS than among *S. aureus* episodes (*p* = 0.08), whereas resolution rates were similar (*p* = 0.16).

**Table 1:** Summary of Patient Characteristics at Baseline (*n* = 56).

	Frequency	%
**Age (years)**		
≤20	4	7.2
21–40	12	21.4
41–60	20	35.7
>60	20	35.7
**Male gender**	23	41.1
**Presence of diabetes**	28	50
**Educational Level**		
Elementary	30	53.6
Secondary	8	14.3
Higher	6	10.7
Illiterate	7	12.5
Unknown	5	8.9
**PD modality**		
APD	9	16.6
CAPD	47	83.3

PD, peritoneal dialysis; APD, automated peritoneal dialysis; CAPD, continuous ambulatory peritoneal dialysis.

**Table 2:** Clinical findings in *S. aureus* peritonitis episodes.

Sign or symptom	N	%
Cloudy Dialysis Effluent	60	96.8
Abdominal pain	42	67.7
Nausea or vomiting	26	41.9
Fever	18	29.0
Hypotension	12	19.3

**Figure 2:**
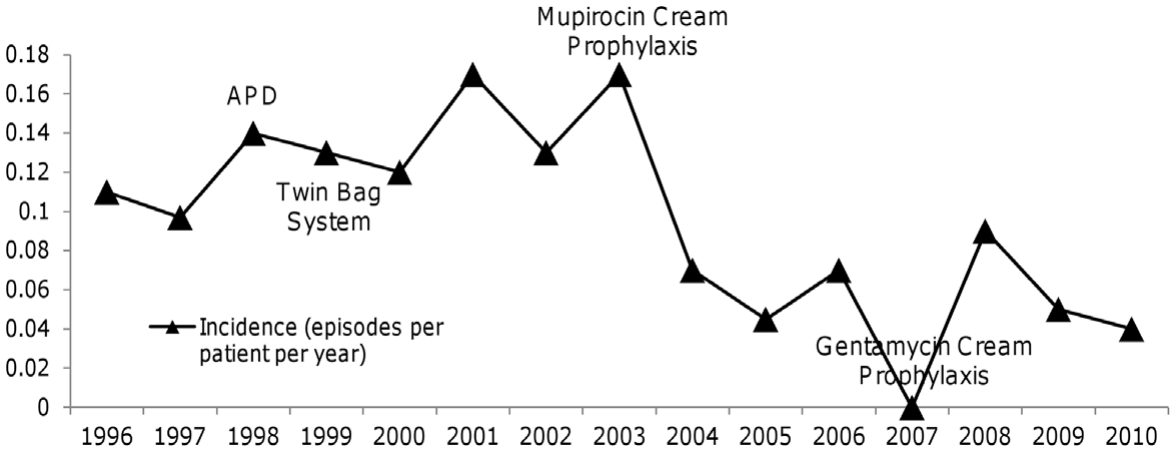
Vancomycin Minimum Inhibitory Concentration (MIC50) and Proportion of Oxacillin-Resistant *Staphylococcus aureus* Strains Isolated from Peritoneal Dialysis Patients with Peritonitis between 1996 and 2010.

All strains were susceptible to vancomycin (MIC≤3 µg/ml) and seven (11.3%) were resistant to oxacillin (MIC≥4 µg/ml). The vancomycin MIC or proportion of oxacillin-resistant isolates did not change significantly over time (Figure 2). The *mec*A gene was detected in seven (11.3%) strains.

The rates of toxin and enzyme production by *S. aureus* are shown in Table 3. No associations were observed between the production of virulence factors and the frequency of initial clinical findings. However, fever was observed in 83.3% of episodes caused by bacteria expressing the *mec*A gene, whereas this clinical symptom was present in only 24% of episodes due to *mec*A gene-negative strains (*p* = 0.03). In addition, there was a trend towards a higher rate of abdominal pain (100%) among strains expressing the *mec*A gene compared to *mec*A gene-negative isolates (64.8%) (*p* = 0.08). The production of virulence factors and presence of the *mec*A gene were not associated with catheter removal, hospitalization, or death rate.

**Table 3:** Production Rates of Pathogenic Factors by *S. aureus* strains Isolated from 62 Peritonitis Episodes.

	N	%
**Enzymes**		
α-Hemolysin	27	43.5
β-Hemolysin	24	38.7
Lipase	52	83.9
Lecithinase	57	91.9
Deoxyribonuclease	58	93.5
Thermonuclease	56	90.3
**Toxins**		
SEA	7	11.3
SEB	17	27.4
SEC	12	19.4
TSST-1	17	27.4
**Biofilm**	55	88.7

SEA, SEB, SEC, enterotoxins A, B and C, respectively; TSST-1, toxic shock syndrome toxin-1.

Gender, age, vancomycin use, presence of diabetes, production of virulence factors (β-hemolysin, lecithinase, deoxyribonuclease, SEC, and TSST-1), presence of the *mec*A gene, and dialysis vintage were associated with a higher chance of non-resolution in univariate analysis (Table 4), and were therefore included in the multivariate logistic regression model. Multivariate analysis showed that the presence of diabetes and β-hemolysin production were factors independently associated with a higher odds ratio of non-resolution of peritonitis episodes. In contrast, age of 41–60 years was associated with a lower chance of non-resolution when compared to age >60 years. No significant association with peritonitis outcome was observed for the other variables (Table 4).

**Table 4:** Odds Comparison of Peritonitis Resolution by Logistic Regression Analysis.

Factor	*p* value(univariate)	*p* value(multivariate)	OR	95% CI
**Gender (female)**	0.109	0.143	4.551	0.599–34.611
**Caucasian race**	0.642			
**Age (years)**	0.042			
≤20		0.999	Exp(3.849)	0.000-
21–40		0.871	0.845	0.111–6.466
41–60		0.020	0.091	0.012–0.684
>60 (reference)				
**Educational level**	0.934			
Elementary				
Secondary				
Higher				
Illiteracy				
**Vancomycin use**	0.037	0.242	0.325	0.049–2.140
**Presence of diabetes**	0.042	0.009	14.682	1.960–112.676
**Enzyme production**				
α-Hemolysin	0.632			
β-Hemolysin	0.077	0.006	16.597	2.246–122.615
Lipase	0.204			
Lecithinase	0.185	0.697	2.248	0.038–131.697
Deoxyribonuclease	0.033	0.999	Exp(6.296)	0.000-
Thermonuclease	0.934			
**Presence of ** ***mec*** **A gene**	0.195	0.838	1.430	0.046–44.342
**Biofilm production**	0.623			
**Toxin production**				
SEA	0.265			
SEB	0.312			
SEC	0.071	0.217	5.621	0.363–87.052
TSST-1	0.205	0.399	0.365	0.035–3.805
**Dialysis vintage (months)**	0.07	0.092	0.943	0.880–1.010
**Dialysis modality** **(APD vs CAPD)**	0.477			

SEA, SEB, SEC, enterotoxins A, B and C, respectively; TSST-1: toxic shock syndrome toxin-1; APD, automated peritoneal dialysis; CAPD, continuous ambulatory peritoneal dialysis.

## Discussion

The present results showed a marked decline in the incidence and proportion of peritonitis episodes caused by *S. aureus* over the past 15 years, in agreement with other studies [3], [12]. The introduction of safer connection systems and the routine use of prophylactic antibiotics at the catheter exit site possibly contributed to the reduction of the incidence of *S. aureus* peritonitis; however the strong decline in the incidence observed after the introduction of the prophylaxis with mupirocin reinforces the role of this strategy on *S. aureus* peritonitis prevention. In addition, in the present series we observed a higher death rate among *S. aureus* episodes compared to episodes caused by CoNS as previously reported [1].

There are few studies reporting the influence of demographic and clinical factors on the prognosis of *S. aureus* peritonitis episodes. Szeto et al. [7] observed an association between adjuvant rifampicin treatment and a significantly lower risk of relapse, whereas the complete cure rate was similar for cephalosporin and vancomycin empiric treatment protocols. Govindarajulu et al. [6] found that the presence of peripheral vascular disease and the use of vancomycin compared to cephalosporins were significantly associated with an increased risk of relapse of *S. aureus* peritonitis. According to these authors, female gender and middle tertile of age were independent predictors of a lower risk of relapse. Similarly, we observed that patient age of 41 to 60 years was associated with a higher chance of peritonitis resolution when compared to older patients. In addition, the presence of diabetes was an independent predictor of non-resolution of peritonitis. It is known that the immune response is dysregulated in diabetic patients, increasing the risk of developing infection. In addition, advanced glycation end-products act on peritoneal mesothelial cells, with a potentially negative impact on the local immune response [13]. Although diabetes has been reported to be a risk factor for peritonitis [14], to our knowledge, there are no studies showing diabetes to be a predictor of poor outcome after a peritonitis episode. In the present series, vancomycin use was not an independent predictor of outcome, in agreement with the study of Szeto et al. [7]. We observed no influence of other demographic or clinical factors on resolution rate. Similar results have been reported by Krishnan et al. [15] in a retrospective series of peritonitis episodes of different causes.

Little is known about the influence of specific virulence factors on peritonitis caused by *S. aureus*. MRSA peritonitis has been associated with poor outcome in the two largest series of *S. aureus* peritonitis [6], [7]. In the present series, we found a low oxacillin resistance rate, which was confirmed by determination of the *mecA* gene. On the other hand, the *S. aureus* strains studied presented expressive enzyme, toxin, and biofilm production. In contrast to previous studies we found no association between oxacillin resistance and resolution rate; however, in the present series only seven peritonitis episodes were caused by oxacillin-resistant *S. aureus*, a fact that may have influenced the results. Episodes caused by *mecA*-positive *S. aureus* isolates were associated with more severe initial clinical symptoms. Studies investigating the role of the *mecA* gene in the virulence of *S. aureus* are scarce in the literature. Fowler Jr et al. [16] found an increasing proportion of MRSA among strains isolated from nasal carriage, uncomplicated bacteremia, and bacteremia with hematogenous complications. Analyzing the same sample later, Gill et al. [17] confirmed a higher frequency of the *mecA* gene among *S. aureus* strains isolated from severe infections. In our laboratory [18] analyzing 336 MRSA and 107 MSSA strains, we observed a significantly higher proportion of strains expressing SEA, SEB, SEC and TSST-1 genes among MRSA. Taken together, these findings show that, although the number of strains expressing the *mec*A gene was small in this series, a role of the *mec*A gene in *S. aureus* virulence cannot be ruled out.

Among bacterial factors studied, β-hemolysin production was significantly and independently associated with lower resolution odds. The role of β-hemolysin in the pathogenesis of *S. aureus* infections has not been previously reported in PD-related peritonitis. Nevertheless, some pathways may be suggested based on the findings of experimental models. β-Hemolysin is one of the toxins produced by *S. aureus* which acts as a sphingomyelinase, degrading sphingomyelin in the outer layer of cell membranes [19]. Deletion of the catalase and β-toxin genes in *S. aureus* strains has been shown to cause strong attenuation of virulence in intramammary and subcutaneous experimental infections of ewes and lambs and in a murine skin abscess model [19]. Using a mouse model of lung injury, Hayashida et al. [20] found that animals infected with β-hemolysin-deficient *S. aureus* presented significantly attenuated lesions compared to those infected with *S. aureus* expressing this toxin. This experimental disease was characterized by intense neutrophilic inflammation and reduced expression of syndecan-1 in alveolar epithelial cells and could be reproduced by administration of recombinant β-hemolysin, but not of mutant β-hemolysin deficient in sphingomyelinase activity.

Extracellular DNA is a major structural component in the biofilms of pathogenic *S. aureus*. Huseby et al. [21] showed that β-hemolysin forms covalent cross-links to itself in the presence of DNA, irrespective of sphingomyelinase activity, producing an insoluble nucleoprotein matrix in vitro. Using an infectious endocarditis rabbit model, the authors observed that this toxin stimulates biofilm formation in vivo. β-Hemolysin does not lyse most types of host cells but leaves them vulnerable to a number of other lytic agents, such as α-hemolysin and Panton-Valentine leukocidin [20]. We recently demonstrated that α-hemolysin production predicts poor outcome in peritonitis episodes caused by *S. aureus* and CoNS [11].

A reservoir of phospholipids exists on the peritoneal surface and the main constituents of peritoneal phospholipids are phosphatidylcholine and sphingomyelin [22]. Indeed, evidence suggests that the phospholipids present on the peritoneal surface are derived from peritoneal mesothelial cells. In this respect, β-hemolysin, a sphingomyelinase, may participate directly in biofilm formation, contributing to a poorer outcome of peritonitis episodes, or may render host peritoneal cells susceptible to other pathogenic factors.

Surprisingly, biofilm production was not a predictor of peritonitis resolution. However, the percentages of non-producers was low, a fact impairing the comparison with biofilm producers; therefore, a role of biofilm production in peritonitis outcome cannot be ruled out. Finally, other *S. aureus* virulence factors that act in a synergistic and coordinated fashion may play a pathogenic role.

The present study has several limitations, the most important of them is the absence of more accurate tests to assess production of β-hemolysin such as mRNA levels using quantitative real time-PCR or specific detection such as by ELISA or Western Blot. Also, the small number of cases analyzed that may reduce its statistical power, and since it is a single-center study its results cannot be extrapolated. Nevertheless, it is the first Latin American study analyzing a series of *S. aureus* peritonitis cases. In this respect, *S. aureus* remains the most frequent PD-related etiology in several Latin American countries, including Brazil [3]. Furthermore, this study focused on the role of virulence factors on the outcome of this infection.

In conclusion, despite a strong reduction in the incidence of *S. aureus* peritonitis, our results showed a poorer outcome of episodes caused by this bacterium when compared to episodes due to CoNS, particularly a higher death rate. Among demographic factors, older age and diabetes were predictors of a lower resolution rate. These findings highlight the importance of peritonitis as a serious complication of PD, particularly among elderly and diabetic patients. β-Hemolysin production was the only virulence factor that negatively influenced peritonitis outcome; however further studies using specific tests to detect the presence of β-hemolysin are necessary to confirm this result.

## Materials and Methods

All episodes of ambulatory PD-related peritonitis caused by *Staphylococcus aureus* between January 1996 and December 2010 were reviewed. The diagnosis of peritonitis was made when at least two of the following criteria were present: 1) presence of a cloudy peritoneal effluent; 2) abdominal pain; 3) dialysate containing more than 100 white blood cells per µl (at least 50% polymorphonuclear cells), and 4) positive culture of peritoneal effluent [23], [24]. Exclusion criteria were episodes of relapsing *S. aureus* peritonitis, presence of concomitant exit site or tunnel infections, and incomplete clinical data. Resolution was defined as the disappearance of signs and symptoms within 96 h after the beginning of antibiotic therapy and a negative peritoneal fluid culture at least 28 days after treatment completion [23], [24]. Relapse was defined as an episode due to the same organism, or a negative culture result that occurs within 28 days of completion of antibiotic therapy for a prior *S. aureus* episode [23], [24]. Death related to peritonitis was defined as death of a patient with active peritonitis, or admitted with peritonitis, or within 2 weeks of a peritonitis episode [23], [24]. Non-resolution was the term used for cases presenting initial non-resolution, relapse, peritoneal catheter removal due to refractory peritonitis, need for a second antibiotic regimen, or death.

The following information was recorded for each case: 1) episode: date, clinical findings, treatment, and outcome (resolution, relapse, catheter removal, or death); 2) presence of diabetes mellitus; 3) demographic data: age, gender and race (Caucasian, non-Caucasian), and dialysis treatment time; 4) dialysis modality (continuous ambulatory peritoneal dialysis or automated peritoneal dialysis); 5) educational level (illiterate, elementary, secondary, and higher).

The study was approved by the Research Ethics Committee of the Faculty of Medicine of Botucatu, Brazil (OF. 028/08-CEP). This study was exempted from the requirement to obtain written informed consent from the participants and/or their legal guardians because the *Staphylococcus* strains included in the study had already been isolated and stored in the Culture Collection of the Department of Microbiology and Immunology, UNESP, Botucatu, São Paulo, Brazil.

Patients were treated within 24 h of the onset of the first clinical signs or symptoms using contemporary empiric antibiotic recommendations [23]–[25]. From 1996 to 2000 (period 1) empiric antibiotic therapy consisted of intraperitoneal (i.p.) cefazolin plus amikacin. Two protocols were used from 2000 to 2005 (period 2): the first consisted of i.p. cefazolin plus amikacin and the second of i.p. cefazolin plus ceftazidime. After 2005 (period 3) all episodes were first treated with i.p. vancomycin plus amikacin. Therapy was evaluated and adjusted as soon as the culture results were available. The duration of antibiotic therapy was 21 days.

In the period 1 no antibiotic prophylaxis at exit site was prescribed and two connection systems were used: the Y set system and the twin bag system, which was introduced in 1999; automated PD (APD) was used from 1998. In the period 2 no antibiotic prophylaxis at exit site was prescribed until 2003, when daily mupirocin cream application at exit site began to be recommended; the twin bag system or APD were prescribed for all patients. In the period 3 until December 2006 all patients were oriented to daily mupirocin cream application, and from January 2007 daily gentamicin cream application at exit site was prescribed to all incident patients; the twin bag system or APD were prescribed for all patients.

The incidence of *S. aureus* peritonitis was calculated for the three subsequent periods of 5 years and is expressed as episodes per patient per year.

The initial cultures were performed with the Bactec® Automated System (Becton Dickinson Company, Sparks, Maryland, USA) and then seeded onto blood agar. The isolates were Gram stained to confirm purity and to determine morphology and specific color. After confirmation of these characteristics, tests for identification of the isolates were performed as recommended by Koneman et al. [26]. The isolates were stored in a culture collection.

The in vitro susceptibility of *S. aureus* to oxacillin and vancomycin was evaluated based on the minimum inhibitory concentration determined by the E-test (AB Biodisk, Solna, Sweden). This quantitative method uses a transparent strip of inert plastic that contains drug concentrations ranging from 0.002 to 256 µg/ml. The proportion of strains susceptible to each drug was defined based on the 2011 CLSI breakpoints [27]. Strains presenting intermediate values were considered to be resistant.

Whole nucleic acids were extracted from *S. aureus* strains cultured on blood agar, individually inoculated into brain heart infusion (BHI) broth, and incubated at 37°C for 24 h. Nucleic acids were extracted using the illustra blood genomic Prep Mini Spin kit (GE Healthcare, Little Chalfont, Buckinghamshire, UK) according to manufacturer instructions. Staphylococcal cells were first digested with lysozyme (10 mg/ml) and proteinase K (20 mg/ml). Next, 500 µl extraction solution was added to the mixture. After centrifugation at 5000 *g* for 1 min, the supernatant was transferred to a GFX column and centrifuged at 5000 *g* for 1 min. The supernatant was discarded and 500 µl extraction solution was added to the column. After centrifugation and disposal of the supernatant, 500 µl wash solution was added to the column. The column was then centrifuged at 14,000 rpm for 3 min and transferred to a 1.5-ml tube. Milli-Q water (200 µl) preheated to 70°C was used for elution. The isolates were centrifuged at 5000 *g* for 1 min and the GFX column was discarded. Extracted DNA was stored under refrigeration at 4°C.

PCR amplification was performed in 0.5-ml microcentrifuge tubes containing 10 pmol of each primer, 2.0 U Taq DNA polymerase, 100 µM deoxyribonucleotide triphosphates, 10 mM Tris-HCl (pH 8.4), 0.75 mM MgCl_2_, and 3 µl nucleic acid in a total volume of 25 µl. Gene *mec*A amplification was carried out in an appropriate thermal cycler using the mecA1 (AAA ATC GAT GGT AAA GGT TGG) and mecA2 (AGT TCT GCA GTA CCG GAT TTG) primers as described by Murakami et al. [28]: 40 cycles of denaturation at 94°C for 30 s, annealing of primers at 55.5°C for 30 s, and extension at 72°C for 1 min. After completion of the 40 cycles, the tubes were incubated at 72°C for 5 min and then cooled to 4°C. The *S. aureus* ATCC 33591 and ATCC 25923 references strains were included in all reactions as positive and negative controls, respectively.

The efficiency of amplification was monitored by electrophoresis on 1.5% agarose gel prepared in 1× TBE buffer and stained with ethidium bromide. The size of the amplified products was compared with a 100-bp standard and the gels were photographed under UV transillumination.

Biofilm production was evaluated according to Christensen et al. [29]. Colonies isolated on blood agar were inoculated into tubes measuring 12.0×75.0 mm and containing 2.0 ml trypticase soy broth and incubated at 37°C for 48 h. Next, 1.0 ml 0.4% trypan blue or Toluidine blue O solution was added to the tubes. After gentle shaking to guarantee staining of the material adhered to the inner surface of the tubes, the dye was discarded. A positive result was defined as the presence of a layer of stained material adhered to the inner wall of the tube. The presence of a colored ring only at the liquid-air surface was classified as a negative result.

Production of α- and β-hemolysin were determined on plates containing blood agar supplemented with 5% rabbit blood and 5% sheep blood, respectively. The plates were incubated at 37°C for 24 h. The formation of hemolysis zones around the isolated colonies indicated a positive result.

Lipolytic activity was evaluated on plates containing blood agar enriched with 0.01% CaCl_2_∶2H_2_O and 1% Tween 80. A positive result was defined as the formation of opacity around the colony after incubation at 37°C for 18 h, followed by incubation at room temperature for 24 h [30]. The production of lecithinase was evaluated using Baird-Parker medium. The formation of an opaque halo around the colony indicated a positive result [31].

Nuclease (DNAse) and thermonuclease (TNAse) were determined by the metachromatic Toluidine blue O agar diffusion-DNA technique according to Lachica et al. [32]. Supernatants obtained by the sac culture method of Donnelly et al. [33] as described below were transferred to the wells of plates containing metachromatic Toluidine blue O agar. The culture supernatant was first heated in a water bath for 20 min for the detection of TNAse. Nuclease (DNAse and TNAse) activity was evaluated by measuring the diameter of pink halos (mm) formed on the medium. Positive results were interpreted by comparing the halos with those obtained for a standard DNAse- and TNAse-positive *S. aureus* strain (ATCC 25923).

For the evaluation of the production of enterotoxins and TSST-1, the sac culture method [33] was used to determine the toxigenic profile of the strains. Dialysis sacs filled with 50 ml double-concentrated BHI broth were placed in U-shaped Erlenmeyer flasks and autoclaved at 121°C for 15 min. A loopful of organisms was added to 18 ml sterile 0.2 M phosphate buffer in 0.9% NaCl, pH 7.4. After incubation at 37°C for 24 h on a shaker at 200 rpm, the cultures were centrifuged at 8000 *g* for 10 min at 4°C and the supernatants obtained were stored at −20°C until the time of use. The extracellular products were detected by reverse passive latex agglutination (RPLA) using the SET-RPLA-T900 and TST-RPLA-TD940 kits (Oxoid Diagnostic Reagents, Cambridge, UK) for the detection of enterotoxins A (SEA), B (SEB), C (SEC) and D (SED) and TSST-1, respectively, according to manufacturer instructions. Samples that presented nonspecific reactions after this treatment were filtered through a Millipore membrane (0.22 µm) and, if necessary, diluted 1∶10 with 0.02 M phosphate buffer in 0.9% NaCl, pH 7.4. A positive reaction was classified as (+), (++) and (+++) according to the agglutination pattern described by the manufacturer of the kit. The formation of a rose button was interpreted as a negative result.

### Statistical analysis

Peritonitis incidences were compared using the Poisson regression model. The association between microbiological characteristics (oxacillin resistance, presence of *mec*A gene and production of pathogenic factors) and the frequency of clinical findings at initial presentation (abdominal pain, fever, nausea or vomiting, and arterial hypotension) was analyzed by the chi-square or Fisher's exact test. These tests were also used to compare resolution and death rates between *S. aureus* peritonitis episodes and 117 contemporary CoNS cases. Multivariate analysis by logistic regression was used to test baseline demographic, clinical, and microbiological factors that independently predicted the outcome of a peritonitis episode. Outcome was classified as two mutually exhausted and exclusive results (resolution or non-resolution). For this purpose, univariate analysis using the chi-square or Fisher's exact test (binary variables) or logistic regression (continuous variables) was first performed to select the variables that would enter the final model, with *p*>0.20 being used as an elimination criterion. A *p* value less than 0.05 was considered to be significant. All statistical analyses were performed using the SPSS 16.0 software (SPSS®, Inc.).
