# Correction: Peritoneal Dialysis-Related Peritonitis Due to *Staphylococcus aureus*: A Single-Center Experience over 15 Years

**DOI:** 10.1371/annotation/6ce124f8-e74f-42dc-81a6-7f85cc23fd3d

**Published:** 2012-05-07

**Authors:** Pasqual Barretti, Taíse M. C. Moraes, Carlos H. Camargo, Jacqueline C. T. Caramori, Alessandro L. Mondelli, Augusto C. Montelli, Maria de Lourdes R. S. da Cunha

There was an error in the placement of Figures 1 and 2. The Figures were swapped and their legends are incorrect. 

